# Letter to the Editor on “Comparative performance of fully-automated and semi-automated artificial intelligence methods for the detection of clinically significant prostate cancer on MRI: a systematic review”

**DOI:** 10.1186/s13244-023-01520-8

**Published:** 2023-11-03

**Authors:** Alessandro Bevilacqua, Margherita Mottola

**Affiliations:** 1 Department of Computer Science and Engineering (DISI), University of Bologna, Bologna, Italy; 2 Advanced Research Center On Electronics Systems (ARCES), University of Bologna, Bologna, Italy; 3 Department of Medical and Surgical Sciences (DIMEC), University of Bologna, Bologna, Italy

**Keywords:** Artificial intelligence, Machine learning, Prostate cancer

## Dear Editor,

We have read the article entitled “Comparative performance of fully-automated and semi-automated artificial intelligence methods for the detection of clinically significant prostate cancer on MRI: a systematic review” by Sushentsev et al. [1], recently published in *Insights into Imaging*, which also mentions our recent publication entitled “The primacy of high B-value 3 T-DWI radiomics in the prediction of clinically significant prostate cancer” [2]. In their comparative review, the Authors address several state-of-art research studies employing Magnetic Resonance Imaging (MRI) and exploiting deep learning and machine learning methods for predicting clinically significant prostate cancer (csPCa). Accordingly, our work is cited because we compare the predictive performance achieved with b2000 Diffusion-Weighted Imaging (DWI_b2000_) and Apparent Diffusion Coefficient (ADC) MRI sequences to classify csPCa and non-csPCa (ncsPCa), finally stating the primacy of DWI_b2000_, that provides by far the best results.

Unfortunately, by reading the work by Sushentsev et al., we have come across many inaccuracies and even errors when referring to either methodology or results of our study, which disqualify our work making it appear as if it had a poor methodological rigour and worse predictive performance than it has.

For this reason, with this letter, we demand that these errors are made public so to recover the integrity of our work.

In the following, we report the errors we detected and, for each of them, we provide the correction. For the sake of clarity, each Table number hereby reported refers to Table in the work by Sushentsev et al.

## Table 1

The Authors present the result of the risk-of-bias assessment analysis performed through the Quality Assessment of Diagnostic Accuracy Studies (QUADAS-2) tool [3] and, exploiting their own protocol developed on purpose for answering each signalling question that regards PCa. As a consequence, our study by Bevilacqua et al. is assigned a “high risk of bias”, arising from an issue related to “Flow and Timing” (i.e., column 4), according to the QUADAS-2 risk system. According to the “Background document” of QUADAS-2, the studies receive a “high risk of bias” if at least one out the three answers (here related to the “Flow and Timing” domain signalling questions) is “NO”. We have got “YES” to two of them while at the third question reported in the Authors’ Additional File 1, that is “biopsy performed at least 6 months before or within 6 months after MRI”, the authors assigned NO, this consequently yielding the “high risk of bias”. The Background document of QUADAS-2 at page 7 states: “Ideally results of the index test and reference standard are collected on the same patients at the same time”.

That is, the nearer, the better. It might be that the Authors meant “at most” instead of “at least”. Anyway, our study considers as the clinical standard the TRUS biopsy “performed six weeks before MRI” examinations. This is fully compliant with QUADAS-2 criteria and the answer should be “YES” instead, this resulting in a “low risk of bias” assigned to our study.

## Table 4

### Column “Discriminative features”

The Authors report “Intensity” as the typology of generated radiomic features. We never mention the intensity, while the features have to be referred to as “First Order” features.

### Column “Feature used for training”

The Authors report “10” as the number of radiomic features exploited to train the classifier, while we used 2 of them.

## Table 5

### Column “PPV”

The authors report “NR”, which means not reported, as regards the Positive Predictive Value (PPV), used for evaluating the classifier predictive performance. Actually, we reported PPV = 0.90 for the DWI_b2000_ model.

### Column “Threshold”

The authors report 0.58 as a threshold value for our study. From their main text, it is not clear what the Authors mean by “Threshold” when they write at page 10: “Specific threshold for diagnostic performance with the resulting characteristics summarised in Table 5”. We guess that they refer to the threshold of the radiomic score between csPCa and ncsPCa groups, and if so, our score was normalized to have threshold equal to 0. Nevertheless, the value reported in Table 5 is right the Youden Index (not a threshold) of our ADC model, while in Table 5 all values refer to our DWI_b2000_ model, which has a Youden Index 0.65. What sounds strange is that under the same column “Threshold” there are negative values, which cannot refer to the Youden Index, that is positive only. Ultimately, this inconsistency remains unsolved.

### Columns “Accuracy” and “NPV”

The authors report “NR”. Actually, although we did not explicitly report accuracy and negative predictive value (NPV) of the holdout test set, since they were not meaningful to the aim of our discussion, we provided all data referring to, or derived from, the contingency table, that is total positive (P) = 20, total negative (N) = 8, false positive (FP) = 2, and false negative (FN) = 2, from which can be easily derived the following values:

$$\mathrm{Accuracy}= \frac{\mathrm{P}+\mathrm{N}-\mathrm{FN}+\mathrm{FP}}{\mathrm{P}+\mathrm{N}}=86\mathrm{\%}$$

$$\mathrm{NPV}= \frac{\mathrm{N}-\mathrm{FP}}{\mathrm{N}-\mathrm{FP}+\mathrm{FN}}=75\mathrm{\%}$$

In conclusion, we are aware that writing a review is a challenging task, for authors, requiring a great amount of work to try standardizing the different information reported, and for reviewers, that somehow need trusting the information extracted from the different cited papers by the authors. With this letter, we aim at providing a useful contribution improving the correctness and the quality of the work by Sushentsev et al., meanwhile restoring the scientific rigour of our research and preserving our reputation.

## Data Availability

All data and material are included in this Letter to the Editor.

## Abbreviations

ADC: Apparent diffusion coefficient
csPCa: Clinically significant prostate cancer
DWI: Diffusion-weighted imaging
FN: False negative
FP: False positive
MRI: Magnetic resonance imaging
N: Negative
NPV: Negative predictive value
ncsPCa: Non-clinically significant prostate cancer
P: Positive
PPV: Positive predictive value
QUADAS-2: Quality Assessment of Diagnostic Accuracy Studies
TRUS: Trans-rectal ultrasound

## References

[1] Sushentsev N, Moreira Da Silvia N, Yeung M (2022). Comparative performance of fully-automated and semi-automated artificial intelligence methods for the detection of clinically significant prostate cancer on MRI: a systematic review. Insights Imaging.

[2] Bevilacqua A, Mottola M, Ferroni F (2021). The primacy of high B-Value 3T-DWI radiomics in the prediction of clinically significant prostate cancer. Diagnostics (Basel).

[3] QUADAS-2. Bristol Medical School: Population Health Sciences. University of Bristol. https://www.bristol.ac.uk/population-health-sciences/projects/quadas/quadas-2/. Accessed on 5 Apr 2022

